# Does age play a role in fever and neutropenia events and complications: A comparison of adolescents versus younger children with cancer at a tertiary care pediatric hospital, a pilot project

**DOI:** 10.1002/cnr2.1767

**Published:** 2022-12-09

**Authors:** Nardin Kirolos, Ken Tang, Lesleigh S. Abbott

**Affiliations:** ^1^ Division of Hematology/Oncology Children's Hospital of Eastern Ontario Ottawa Ontario Canada; ^2^ CHEO Research Institute Children's Hospital of Eastern Ontario Ottawa Ontario Canada

**Keywords:** adolescents with cancer, fever, neutropenia

## Abstract

**Background:**

Adolescents and young adults with cancer (AYA) are a complex group of patients. The development of fever and neutropenia (FN) is a potentially lethal complication of chemotherapy. Risk stratification of patients with FN has become increasingly valuable allowing for early intervention and to guide treatment type and duration appropriately. There are risk stratification guidelines that exist, but most are validated in young children with cancer (YCWC). AYA are frequently shown to have more numerous and severe side effects from chemotherapy.

**Aims:**

This study aimed to identify whether age contributes to the incidence and severity of FN.

**Methods and Results:**

Patients diagnosed with a malignancy in a 5‐year period at our institution were included from ages 0–18 years. We reviewed details of their FN events, including duration of hospital admission, source (bacterial/fungal), PICU admission and duration, positive blood cultures and mortality. Adolescents with cancer (AWC) had a trend of being 1.56 times more likely to have FN events (CI 95% 0.936–2.622, *p* = 0.087). Assessment of the duration of PICU stay showed that AWC were 4.9 times more likely to have longer admissions (CI 95% 0.998–24.067, *p* = 0.050). There was no significant difference between the two groups in the rate of PICU admission, positive cultures, identification of a bacterial or fungal source, hospital admission duration or mortality from FN.

**Conclusion:**

This study demonstrated a trend towards AWC being more likely to develop FN events. When such events occur in this group, the severity of them may be heightened as evidenced by longer duration of PICU admission.

## 1 INTRODUCTION

Adolescents and young adults with cancer (AYA) are commonly defined as those who fall within the age range of 15–29 years, with certain institutions extending this definition to include those up to 39 years of age.^1^, ^2^ This group faces numerous healthcare challenges that disproportionately impact their overall health outcomes and well‐being compared to their pediatric and adult counterparts with the same diagnoses.^1^, ^2^ Some of these disparities in care arise from their unique biologic, epidemiologic, and psychosocial factors in association with their underrepresentation in clinical trials.^3^, ^4^, ^5^ AYA have additionally been noted to suffer more side effects from therapy compared to younger children.^5^, ^6^ Additional consideration must also be given to the discontinuity of care faced by AYA who are often treated at pediatric institutions until 18 years of age then transitioned to adult institutions beyond this, creating both physical and academic barriers to studying this population. Given the varying treatment protocols and approach to the management of complications between adult and pediatric institutions, the treatment of AYA is overall less uniform. All these aspects have contributed to the lag in improvement in cancer mortality for the AYA population, in contrast to the more drastic improvements made in younger children with cancer (YCWC).^5^, ^6^ These unique factors highlight the need for further recognition of cancer associated risks in order to limit adverse health outcomes and improve overall survival for this group, by creating more standardized treatment guidelines.^3^, ^4^, ^5^

A common oncologic emergency is fever and neutropenia (FN) which is associated with increased morbidity and mortality.^7^, ^8^, ^9^ The presence of neutropenia can result in an increased risk of life‐threatening infections and chemotherapy dose reductions and delays, which may negatively impact treatment outcomes.^10^, ^11^ Given that about half of the pediatric oncologic population develop at least one episode of FN when treated with chemotherapy, routine emergency hospitalization upon presentation and empirical antibiotic treatment has significantly reduced mortality over the years to around 1%.^12^ However, FN presentations vary among patients with delayed recognition resulting in worse outcomes prompting the need for improvements in patient risk assessments.^13^, ^14^

Risk stratification strategies and clinical decision rules for presentations of FN have been adopted internationally in order to provide appropriate individualized treatment for patients.^15^ Various risk factors have been identified in predisposing patients to the development of FN, some of which include chemotherapy type and dose intensity, advanced age, comorbidities, elevated C‐reactive protein, hypotension, leukemia as the cancer type, thrombocytopenia, and acute malnutrition.^16^, ^17^ For patients who meet criteria of low risk, a reduced intensity treatment regimen as an outpatient, has been shown to be safe, improve quality of life and reduce costs of care.^18^ However, such stratification guidelines are based on young pediatric patients, rather than AYA and even within such regulations there remains an ongoing uncertainty as to the safety and efficacy of their applicability in all clinical settings.^18^, ^19^

Due to the uncertainty of these risk indices, treatment of FN with intravenous antibiotics, irrespective of underlying risk, is most common.^20^, ^21^ Further investigations are required to identify the feasibility of using risk factors, such as patient age, to risk stratify patients presenting with FN in order to further advance management, risk assessment and even consideration of preventative measures for cancer patients with this risk factor.

The main objective of this study is to assess the difference in quantity of FN presentations and severity between adolescents with cancer (AWC) and YCWC who present to our pediatric institution. Given that our institution only treats patients up to 18 years of age, our study group of focus will be the AWC. The results of this study will be particularly pertinent to pediatric oncology clinicians who typically treat up to 18 years of age in their institutions.

The primary aim of this retrospective chart review was to compare the proportion of AWC to the proportion of YCWC who experienced FN following initial malignancy diagnosis to determine whether age is a risk factor for FN events. The secondary objective of this study was to identify the differences in severity of FN events in AWC and YCWC based on secondary variables including the duration of hospitalization, incidence of a bacterial or fungal cause of the event, incidence of a resulting PICU admission, duration of PICU admission, incidence of positive blood cultures and incidence of mortality due to FN.

## 2 METHODS

We conducted a retrospective chart review of all patients diagnosed with a malignancy from January 1, 2013 to January 1, 2018 at the Children's Hospital of Eastern Ontario (CHEO). CHEO is a tertiary care, medium size institution in Ottawa, Canada that treats children from age 0–18 years old. This study was approved by the Research Ethics Board at CHEO. All malignancy types were included in the study, with no age restriction. Patients who presented with a relapse from a previous cancer diagnosis or who did not receive chemotherapy as part of their treatment regimen were excluded as their risk factors for severity and number of episodes of FN were felt to be greater or lesser, respectively.

For each enrolled patient, data on their sex, date of diagnosis, age at the time of diagnosis, primary oncologic diagnosis, treatment protocol(s), date of treatment completion, and current mortality status were collected from their health record. A FN event was identified at any time when the patient presented with an absolute neutrophil count (ANC) of less than 500 cells/mm^3^ and fever according to institutional definition. Fever was defined as an axillary temperature of 38°C or greater measured once or a temperature between 37.6–37.9°C measured twice at timepoints an hour apart. If measured orally, patients were considered febrile at 38.5°C or greater measured once, or a temperature between 38.0–38.4°C measured twice after an hour's time. For each FN episode, information was collected on the date of presentation and age of the patient at that time. Severity was assessed by consideration of our secondary outcomes, which are the duration of hospital admission, the discovery of a source for the FN (bacterial/fungal), a resulting PICU admission and duration, the presence of a positive blood culture and mortality from the FN event. Multiple FN episodes per patient were counted and assessed. The hospital length of stay variable was only calculated based on admissions where patients presented to the emergency department with FN, rather than recorded FN events that developed during a hospital stay for an alternate admitting diagnosis. The PICU admission and length of stay variables were calculated based on admissions to the PICU that were directly related to a FN event. The average length of stay for these variables was calculated with inclusion of a length of stay of zero.

Patient characteristics and outcomes were described using descriptive statistics. Continuous variables were summarized as means, standard deviations, and range, and categorical variables were summarized as frequencies and percentages. Due to the varying treatment durations and therefore the varied risk for the number of FN events during the course of individual patient treatments, the rate of FN was also quantified and described, which was calculated as the total number of each type of FN over the treatment duration in years. Patients were sub‐categorized by age into the AWC if they were 15 years or older up to 18 years old at the time of their cancer diagnosis or in the YCWC group if they had an age of less than 15 at the time of their cancer diagnosis. Quasi‐poisson models were fitted to assess the relationship between age groups and FN complications, while controlling for key covariates of gender and diagnosis type (7 levels, leukemia set as reference category). In these models, the number of FN events was specified as the outcome, while the natural logarithm of treatment duration was specified as an offset parameter. Effect sizes were expressed in terms of adjusted rate ratios and associated 95% confidence intervals (95%CI). All analyses were conducted with R version 4.0.5.^22^

## 3 RESULTS

**FIGURE 1:**
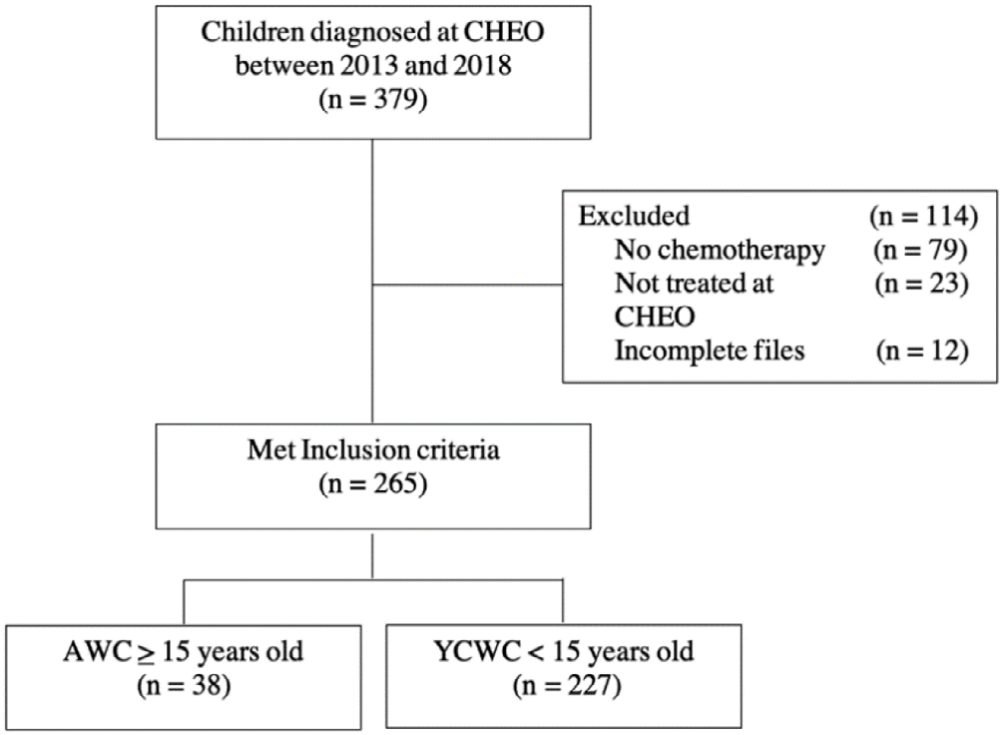
Flow of patients in the study.

Between January 2013 and January 2018, 379 children and adolescents were diagnosed with a malignancy; 265 of which were included in the analysis (Figure 1). Data was collected for each enrolled patient until June 2019. There were 14.3% of patients with a minimum age of 15 years old who were classified under the AWC group. The median age at diagnosis was 6.80 (IQR, 3.2–12.86 years) for all patients, 16.46 (IQR, 15.5–17.38 years) for the AWC and 5.93 (IQR, 2.68–9.88 years) for the YCWC. Over half the patients were male (55.8%). Patients had varying cancer diagnoses of which the majority were leukemias (41.1%), followed by lymphomas (18.5%), primary brain tumors (15.1%), neuroblastoma (6.4%), sarcomas (6.0%), Wilms tumor (4.9%) and germ cell tumors (1.9%). The remaining diagnoses (6.0%) were grouped together due to their rarer occurrence. The proportion of varying diagnoses was significantly different (*p <* 0.001) between the AWC and YCWC groups, with lymphoma (36.8%) predominating in the AWC group and leukemia (39.2%) predominating in the YCWC group. The treatment duration (mean ± SD) for the YCWC of 1.85 ± 1.39 years was significantly longer (*p <* 0.001) than that of the AWC, which was 1.01 ± 0.93 years. Additional demographic and treatment characteristics are summarized in Table 1.

**TABLE 1:** Demographic, disease and treatment characteristics of all patients

	All patients (*N* = 265)	AWC (*n* = 38)	YCWC (*n* = 227)
Median age, years (IQR)	6.80 (3.20, 12.86)	16.46 (15.50, 17.38)	5.93 (2.68, 9.88)
Age group, *n* (%)	265 (100)	37 (14.0)	228 (86.0)
Female, *n* (%)	117 (44.2)	16 (42.1)	101 (44.5)
Diagnosis			
Leukemia (*n*, %)	109 (41.13)	14 (36.84)	95 (41.85)
Lymphoma (*n*, %)	49 (18.49)	14 (36.84)	35 (15.42)
Primary brain tumor (*n*, %)	40 (15.09)	5 (13.16)	35 (15.42)
Neuroblastoma (*n*, %)	17 (6.4)	0 (0)	17 (7.5)
Sarcoma (*n*, %)	16 (6.0)	3 (7.9)	13 (5.7)
Other^a^ (*n*, %)	16 (6.0)	1 (2.6)	15 (6.6)
Wilms Tumor (*n*, %)	13 (4.9)	0 (0)	13 (5.7)
Germ cell tumors (*n*, %)	5 (1.9)	1 (2.6)	4 (1.8)
Mean treatment duration, years (SD)	1.73 (1.37)	1.01 (0.93)	1.85 (1.39)
Deceased (*n*, %)	29 (10.9)	0 (0)	29 (12.8)

Abbreviations: AWC, adolescents with cancer; YCWC, young children with cancer.

^a^ Other diagnoses include eosinophilic granuloma, hepatoblastoma, hemophagocytic lymphohistiocytosis, rhabdoid tumor, langerhans cell histiocytosis, lymphoproliferative disorder, blastic plasmacytoid dendritic cell neoplasm.

**FIGURE 2:**
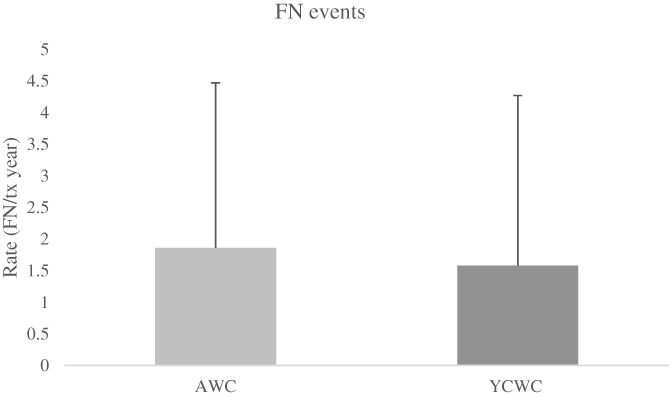
Rate of fever and neutropenia (FN) events in the adolescents with cancer (AWC) and young children with cancer (YCWC). SD, error bars plotted.

The mean frequency and mean rate of FN events and severity markers in the AWC and YCWC groups is summarized in Table 2. In the AWC group, the average FN event occurrence was 1.71 (SD 2.42), with 10.5%, 13.2% and 15.8% of patients having one, two or three FN episodes, respectively. In this group, 47.4% of patients did not have any FN events. The maximum number of 10 episodes was had by one patient (2.6%). In contrast, the YCWC patients had an average of 1.84 (SD 1.91) FN events. In this group, 36.6% of patients did not have any FN events, while the majority of those who had an event had only one episode (16.7%). The remaining patients had two (11.5%) or three (13.7%) episodes. One patient (0.4%) had the maximal number of nine episodes in this age group. The rate of FN among AWC and YCWC was 1.86 ± 2.61 and 1.58 ± 2.69, respectively (Figure 2) yielding an adjusted rate ratio of 1.567 ([CI 95% 0.936, 2.622], *p* = 0.087) for FN events of AWC compared to YCWC, adjusted for covariates of gender and all diagnosis types.

**TABLE 2:** Summary of the mean frequency and mean rate of fever and neutropenia (FN) events and severity markers in the adolescents with cancer (AWC) and young children with cancer (YCWC) groups, where rates are calculated as the mean number of an outcome over the mean treatment duration in years.

	AWC (*n* = 38)		YCWC (*n* = 227)		*P*
	Frequency, mean (SD)	Rate, mean (range)	Frequency, mean (SD)	Rate, mean (range)	
FN episodes	1.71 (2.42)	1.86 (0.00–9.49)	1.84 (1.91)	1.58 (0.00–20.58)	0.087
FN source (Bacterial/Fungal)	0.21 (0.47)	0.21 (0.00–2.95)	0.27 (0.67)	0.20 (0.00–6.19)	0.680
Bacterial	0.16 (0.37)	0.19 (0.00–2.95)	0.27 (0.67)	0.20 (0.00–6.19)	
Fungal	0.05 (0.23)	0.02 (0.00–0.41)	0.00 (0.00)	0.00 (0.00–0.00)	
Positive blood cultures	0.21 (0.47)	0.21 (0.00–2.95)	0.30 (0.68)	0.22 (0.00–6.19)	0.780
PICU admission	0.05 (0.23)	0.07 (0.00–2.30)	0.04 (0.22)	0.03 (0.00–3.10)	0.137

Abbreviations: AWC, adolescents with cancer; FN, fever and neutropenia; PICU, pediatric intensive care unit; YCWC, young children with cancer.

In evaluation of the secondary outcomes for severity of FN episodes, the AWC had an average of 0.16 (SD 0.37) bacterial episodes with only 15.8% of the group having one such documented episode. They averaged 0.05 (SD 0.23) fungal episodes, experienced by only 5.3% of the group. The YCWC group had a mean average of 0.27 (SD 0.67) bacterial episodes, with the majority of those confirmed to have a bacterial infection having only one episode (12.8%), but a minority having two (2.2%), three (2.6%), or four (0.4%) episodes. In the YCWC group, no episodes were related to a fungal infection. In comparing rates of the AWC to YCWC, for the bacterial episodes they were 0.19 (SD 0.61) to 0.20 (SD 0.71) and for the fungal causes they were 0.02 (SD 0.09) to 0.0 (SD 0).

**FIGURE 3:**
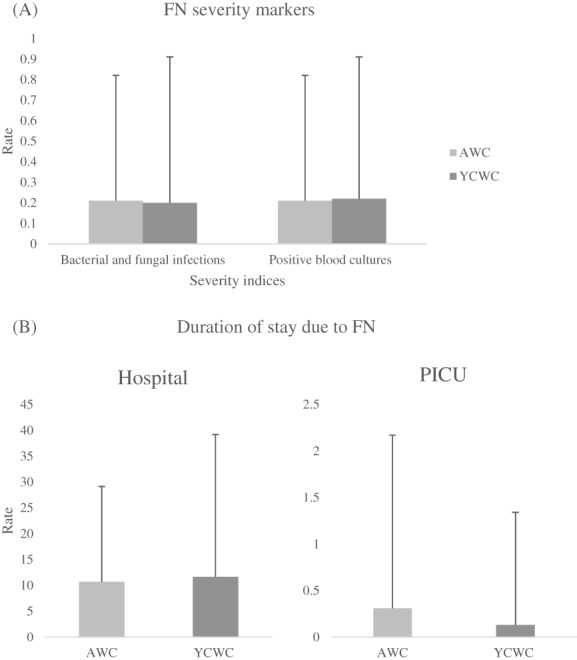
Rate of secondary outcomes of severity of fever and neutropenia (FN) episodes in the adolescents with cancer (AWC) and young children with cancer (YCWC), including (A) identified bacterial and fungal infections in a FN episode and frequency of positive blood cultures (either bacteremia or fungemia) and (B) rate of duration of the hospital and pediatric intensive care unit (PICU) admission in days in those admitted for FN. SD error bars plotted.

When combining the identification of bacterial and fungal sources during all FN episodes, those in the AWC group had an overall average number of 0.21 (SD 0.47), with 18.4% of the patients having at least one of these events. The YCWC had a mean of 0.27 (SD 0.67), with 18.1% of the group having between one and four occurrences of this severity index. The rate for the AWC group was 0.21 (SD 0.61) compared to the YCWC group which was 0.20 (0.71). When collating all the findings to assess for the role that age has on the severity of FN presentations denoted by the identification of bacterial and/or fungal sources, an adjusted rate ratio of 1.258 ([CI 95% 0.423, 3.736], *p* = 0.680) for the AWC compared to the YCWC was found (Figure 3A).

In examination of severity based on the incidence of positive blood cultures, either bacteremia or fungemia, during FN presentations, the AWC group averaged 0.21 (SD 0.47) with 18.4% of the group having 1 or 2 positive blood cultures. The rate of this index was 0.21 (SD 0.61). For the YCWC, they had an average of 0.30 (SD 0.68) positive blood cultures. In the YCWC group, 20.7% of the patients had a minimum of one positive blood culture and the rate for the group was found to be 0.22 (SD 0.69). As such, the adjusted rate ratio for the finding of a positive blood culture in AWC vs. YCWC was 1.155 ([CI 95% 0.421, 3.172], *p* = 0.780) (Figure 3A).

Further severity assessment based on time spent in hospital for patients with the main admitting diagnosis of FN revealed that the AWC spend an average of 10.11 (SD 16.41) days in hospital, while the YCWC spent 14.84 (SD 23.54) days. The AWC had a rate of 10.70 (SD 18.43), while the YCWC had a rate of 11.64 (SD 27.53) (Figure 3B). Comparison of the time spent by both groups yielded an adjusted ratio of 1.136 ([CI 95% 0.536, 2.405], *p* = 0.740).

Evaluation of the severity index of PICU admission revealed that for the AWC, 5.3% of patients in this age group had one PICU admission. In contrast, 3.5% of patients under 15 years of age had at least one PICU admission. Analysis of the duration of PICU admission revealed that the average duration of stay for the entire AWC group in PICU, including those with 0 admission days, was 0.16 (SD 0.82) days with a rate of 0.31 (SD 1.86) and 0.12 (SD 0.97) days for the YCWC with a rate of 0.13 (SD 1.21) (Figure 3B). The adjusted rate ratio of the AWC to the YCWC was statistically significant at 4.900 ([CI 95% 0.998, 24.067], *p* = 0.050). The mean duration in days and mean rate of the hospital and PICU admission durations in the AWC and YCWC groups are summarized in Table 3.

**TABLE 3:** Summary of the mean duration in days and mean rate of the hospital and pediatric intensive care unit (PICU) admission durations in the adolescents with cancer (AWC) and young children with cancer (YCWC) groups. Further frequency counts and percentages are included for the various PICU admission days.

	AWC (*n* = 38)		YCWC (*n* = 227)		*P*
	Days, mean (SD)	Rate, mean (range)	Days, mean (SD)	Rate, mean (range)	
Hospital admission duration	10.11 (16.41)	10.70 (0.00–75.20)	14.84 (23.54)	11.64 (0.00–269.82)	0.740
PICU admission duration	0.16 (0.82)	0.31 (0.00–11.49)	0.12 (0.91)	0.13 (0.00–13.30)	0.050
0 days, freq (%)	36 (94.7)		219 (96.5)		
1 day, freq (%)	1 (2.6)		5 (2.2)		
4 days, freq (%)	0 (0)		1 (0.4)		
5 days, freq (%)	1 (2.6)		1 (0.4)		
13 days, freq (%)	0 (0)		1 (0.4)		

Abbreviations: AWC, adolescents with cancer; PICU, pediatric intensive care unit; YCWC, young children with cancer.

Among all patients enrolled in the study, there was 10.9% of mortality, made up entirely of the YCWC group, but no death was attributable solely to a FN event.

## 4 DISCUSSION

We observed that compared to YCWC, AWC show a trend towards being more likely to develop FN given an adjusted rate ratio of 1.6 for the incidence of FN in AYC to YCWC. Additionally, when such events occur in this age group, there is a trend towards increased severity of FN as shown by the higher rates of PICU admission, with a rate of 0.07 in the AWC group compared to 0.03 in the YCWC group, and longer admission to PICU, when admission is secondary to their FN presentation, as shown through the statistically significant finding of a rate ratio of 4.9 when comparing duration of PICU admission in the AWC to the YCWC.

Given our findings that suggest that age may play a role in FN, and the known harmful effects of neutropenic sepsis, it is imperative that this group of patients is prioritized for their own set of guidelines that account for age in risk stratification.^10^, ^11^ Risk assessment guidelines are essential in establishing a standard for the extent of necessary investigations, type of empirical antibiotic therapy to be used with regards to the formulation in which they are provided, duration of antibiotic therapy, neutrophil recovery necessary prior to discharge or discontinuation of antibiotics, the need for prophylaxis throughout chemotherapy treatment and determination of whether treatment is conducted inpatient or outpatient.^14^

There are numerous risk stratification tools (i.e., Rackoff,^23^ Alexander,^24^ PINDA,^25^ SPOG,^26^ Ammann^27^ and MASCC^28^) that have been developed to better stratify patients as low risk based on the likelihood of severe outcomes as a result of FN. Although there have been numerous studies, such as Haeusler et al., which have validated many of these existing risk stratification tools, the median age of the study patients on which these tools are validated typically falls within the YCWC population with a median age of 5–6 years of age.^18^ Additionally, countless studies have shown that all rules perform inadequately in their ability to appropriately discriminate AYA patients as low or high risk.^19^ The inconsistent performance of these tools across cohorts has left various panels aimed at developing guidance statements to advise that individual sites select which rules to use based on their available resources and population data, rather than evidence based superiority of rules.^29^, ^30^ The findings of this study do not suggest that all previous risk factors studied in the YCWC group be abandoned for the AYA population; they do however support the implementation of age into such existing guidelines in order to individualize them to this unique population.

Our findings which suggest that adolescence as an individual variable may increase both the incidence and severity of FN in the pediatric oncological population provide an opportunity in which age can be used to risk stratify younger patients with cancer to a lower risk group.^19^ Similar approaches to this have been shown to be effective in the adult oncology population who present with FN but are classified as low risk.^31^ These results also bring up the consideration for the use of prophylactic antimicrobials for AWC patients in future research as well as clinically. Further refinement of risk factors could help decrease the incidence of these mentioned events. With the addition of our findings that PICU admission duration in the AWC is significantly longer, early identification and treatment of this age group in particular may not only contribute to decreasing the likelihood of serious complications, but will also be more cost effective for institutions.

Age may play a role in the incidence and severity of FN due to both biological and social characteristics that are distinct to the adolescent population. As our study did not further classify the groups based on cancer diagnoses, but the distribution of varying malignancies did significantly vary between the groups, with lymphomas predominating in the AWC, a finding commonly noted in previous studies,^2^ there is potential that malignancy type could be contributing to the incidence and severity of outcomes, such as FN. Given the varying cancer diagnoses, the chemotherapy regimens are also different and thus pose different complication risks for events such as FN. Future studies can investigate this by comparison of the specific chemotherapy regimens used for YCWC and AWC for the same malignancies to further elucidate whether age is an independent variable in the incidence and severity of FN, or whether its role may be linked to the chemotherapy regimens and their associated risks for FN.

From a social perspective, nonadherence to treatment has long been shown to contribute to lower rates of survival improvement among the AYA group.^32^, ^33^ With the increase in autonomy in this group, there is a greater role for these patients to self‐administer medications such as oral chemotherapy, and in previous studies up to 60% of AYA have been found to fail adherence to recommendations made by their medical team.^34^, ^35^, ^36^ One can consider that similarly this autonomy may lead to later presentations of FN and thus more severe outcomes when they do present. Varying factors have been linked to nonadherence in the AYA group including developmental factors such as underdeveloped coping skills, interpersonal factors such as poor communication with their healthcare team, and educational factors including knowledge of their illness, treatment and side effects.^37^ With this understanding that many barriers exist that can impact adherence and thus clinical outcomes in the AYA population, recommendations are now being made that focus on adherence‐promoting strategies for patients, caregivers and health care providers.^37^ Our findings that age may result in increased FN events in the AWC group further support the need for active implementation of such interventions. In addition to regular oncologic treatments and the potential for age to be used as a risk factor in risk stratification guidelines for FN, our findings highlight the importance of early implementation of these strategies as a preventative approach that could decrease the incidence and severity of FN in AWC.

This study is limited by its retrospective design and the associated restrictions. Additionally, given that CHEO only treats patients to an upper limit age of 18 years old, our study focused on the AWC population, rather than the broader AYA group. In future projects, we plan on working with our adult oncology teams to expand the AWC group to include patients up to 29 years of age, in order to have our findings be more applicable to the entire AYA group. However, our findings are pertinent to the majority of pediatric oncology clinicians who typically treat up to the age of 18 years old at their institutions. Our findings are preliminarily assessing patients with cancer irrespective of their diagnosis or treatment regimen. Future studies may further assess the role of how the known disproportionate incidence of FN with various malignancies and different chemotherapy regimens also contribute either similarly or differently to patients with cancer of varying ages. Information pertaining to patients' organ dysfunction can also aid in providing an alternate explanation for why certain patients, particularly those of the AYA group, may experience increased severity in FN episodes. Additionally, patients' social and demographic characteristics may also further inform on any differences noted between the different age groups.

In conclusion, this study highlights that differences in FN event occurrence and severity exist between AWC and YCWC. Further refinement of risk factors is necessary in order to better risk stratify oncology patients and develop more appropriate, individualized treatment algorithms.

## AUTHOR CONTRIBUTIONS

**Nardin Kirolos:** Data curation (lead); investigation (lead); writing – original draft (equal); writing – review and editing (equal). **Kenneth Tang:** Data curation (equal); formal analysis (lead); methodology (supporting); writing – original draft (equal); writing – review and editing (equal). **Lesleigh S Abbott:** Conceptualization (lead); data curation (supporting); formal analysis (supporting); investigation (supporting); methodology (lead); project administration (lead); resources (lead); supervision (equal); validation (equal); visualization (lead); writing – original draft (equal); writing – review and editing (equal).

## CONFLICT OF INTEREST

The authors have stated explicitly that there are no conflicts of interest in connection with this article.

## ETHICS STATEMENT

This study was approved by the Research Ethics Board at the Children's Hospital of Eastern Ontario.

## Data Availability

The data that support the findings of this study are available on request from the corresponding author. The data are not publicly available due to privacy or ethical restrictions.
